# Clinical surrogates of dysautonomia predict lethal outcome in COVID-19 on intensive care unit

**DOI:** 10.1186/s42466-023-00243-x

**Published:** 2023-05-04

**Authors:** Marcel Seungsu Woo, Christina Mayer, Marlene Fischer, Stefan Kluge, Kevin Roedl, Christian Gerloff, Patrick Czorlich, Götz Thomalla, Julian Schulze zur Wiesch, Nils Schweingruber

**Affiliations:** 1 Department of Neurology, University Medical Center Hamburg-Eppendorf, Martinistraße 52, 20246 Hamburg, Germany; 2 Institute of Neuroimmunology and Multiple Sclerosis (INIMS), Center for Molecular Neurobiology Hamburg (ZMNH), University Medical Center Hamburg-Eppendorf, 20246 Hamburg, Germany; 3 Department of Intensive Care Medicine, University Medical Center Hamburg-Eppendorf, 20251 Hamburg, Germany; 4 I. Department of Medicine, University Medical Center Hamburg-Eppendorf, 20246 Hamburg, Germany; 5 German Center for Infection Research (DZIF), Partner Site Hamburg-Lübeck-Borstel-Riems, 20246 Hamburg, Germany; 6 Department of Neurosurgery, University Medical Center Hamburg-Eppendorf, 20251 Hamburg, Germany

**Keywords:** SARS-CoV-2, Heart rate variability, Autonomic regulation, Dysautonomia, Intensive care unit

## Abstract

**Background:**

Unpredictable vegetative deteriorations made the treatment of patients with acute COVID-19 on intensive care unit particularly challenging during the first waves of the pandemic. Clinical correlates of dysautonomia and their impact on the disease course in critically ill COVID-19 patients are unknown.

**Methods:**

We retrospectively analyzed data collected during a single-center observational study (March 2020–November 2021) which was performed at the University Medical Center Hamburg-Eppendorf, a large tertiary medical center in Germany. All patients admitted to ICU due to acute COVID-19 disease during the study period were included (n = 361). Heart rate variability (HRV) and blood pressure variability (BPV) per day were used as clinical surrogates of dysautonomia and compared between survivors and non-survivors at different time points after admission. Intraindividual correlation of vital signs with laboratory parameters were calculated and corrected for age, sex and disease severity.

**Results:**

Patients who deceased in ICU had a longer stay (median days ± IQR, survivors 11.0 ± 27.3, non-survivors 14.1 ± 18.7, *P* = 0.85), in contrast time spent under invasive ventilation was not significantly different (median hours ± IQR, survivors 322 ± 782, non-survivors 286 ± 434, *P* = 0.29). Reduced HRV and BPV predicted lethal outcome in patients staying on ICU longer than 10 days after adjustment for age, sex, and disease severity. Accordingly, HRV was significantly less correlated with inflammatory markers (e.g. CRP and Procalcitonin) and blood carbon dioxide in non-survivors in comparison to survivors indicating uncoupling between autonomic function and inflammation in non-survivors.

**Conclusions:**

Our study suggests autonomic dysfunction as a contributor to mortality in critically ill COVID-19 patients during the first waves of the pandemic. Serving as a surrogate for disease progression, these findings could contribute to the clinical management of COVID-19 patients admitted to the ICU. Furthermore, the suggested measure of dysautonomia and correlation with other laboratory parameters is non-invasive, simple, and cost-effective and should be evaluated as an additional outcome parameter in septic patients treated in the ICU in the future.

## Background

SARS-CoV-2 targets multiple organ systems resulting in a wide range of symptoms in COVID-19 patients that requires multidisciplinary efforts for clinical management [1]. Dysautonomia is frequently reported by COVID-19 patients. Symptoms include palpitations, orthostatic dysregulation, fatigue or exercise intolerance which is observed in patients during the acute infection but also commonly after recovery from COVID-19 [2, 3] and is included in the Long-COVID symptom complex [4]. First attempts to quantify autonomic dysregulation in COVID-19 patients showed an increased prevalence of orthostatic hypotension that correlated with disease severity [5]. Further characterization and management of autonomic dysfunction are of major interest since it might increase mortality and predict disease severity during the acute phase of infection with SARS-CoV-2 and could explain some of the symptoms experienced by patients suffering from Long-COVID.

Especially during the first waves of the pandemic clinical management of critically ill COVID-19 patients was challenging due to rapid and unpredictable deteriorations [6, 7]. These were characterized by sudden respiratory failure with the need for mechanical ventilation which was often accompanied by disseminated coagulopathy and severe hemodynamic instability [8–11]. Recent studies have shown that, against expectancy, COVID-19 patients with sepsis do not display an adequate increase in heart rate (HR) in comparison to other infectious septic syndromes [12]. However, it is still unclear whether the HR variability in COVID-19 patients uncouples from physiologic modulators such as respiratory hypercapnia or inflammatory parameters and whether dynamic measurements of vital signs could be used to distinguish COVID-19 survivors from non-survivors.

Clinical manifestations of SARS-CoV-2 infection that point towards the involvement of the autonomic nervous system (ANS) are supported by *post-mortem* studies. Thus, SARS-CoV-2 RNA and nucleocapsid were detected in the brainstem and cranial nerves of deceased COVID-19 patients [13, 14]. The main viral entry sites are present in structures within the vagal nerve that express the ACE2 receptor, the TMPRSS2 intracellular protease, and the membrane protein NRP1 [15]. Therefore, it is highly plausible that the occurrence of viral infection harms structures of the ANS directly. In addition, indirect damage to the ANS caused by immune cell infiltration into the central nervous system [16, 17] and systemic production of cytokines [18–20] have been observed. However, the functional consequences and clinical implications of autonomic dysregulation and parasympathetic malfunctions of the cardio-respiratory system are still ill-defined.

In this study, we hypothesized that biomarkers of dysautonomia predict worse clinical outcome of COVID-19 patients who were admitted to ICU. Therefore, we used HRV and BPV as indicators of autonomic function and compared survivors with non-survivors. Furthermore, we tested our hypothesis that dysautonomia in severe COVID-19 leads to uncoupling of physiologic heart rate adaptations by correlating HRV and laboratory parameters of inflammation and respiration. Accurate and timely diagnosis of dysautonomia will guide clinical management and risk stratification of COVID-19 patients for lethal outcome on ICU.

## Methods

### Study design and setting

We performed a retrospective analysis of an observational single-center cohort study. The institutional and interdisciplinary department of intensive care medicine of the university medical center Hamburg-Eppendorf (UKE) operates 140 high-care ICU beds and treats approximately 5700 patients per year. All patients admitted to ICU due to COVID-19 during the study period between March 2020 and November 2021 were included (n = 361).

### Participants and data sources

The ICU is equipped with Dräger® Monitoring systems (Infinity Delta, Lübeck, Germany). Patient information, laboratory values, arterial blood gas samples, and vital parameters are stored centrally in the Dräger® supported Software Integrated Care Manager (ICM). To access a passively saved reporting database ICMiq from Dräger® is applied at the University Medical Center Hamburg-Eppendorf, Germany. All patients admitted to ICU of the university medical center Hamburg-Eppendorf (UKE) due to COVID-19 during the study period (03/2020–11/2021) were included and the infection was confirmed through PCR testing. Underage patients were excluded in this study. Clinical management was performed according to national and international guidelines, including prone positioning in moderate to severe ARDS and restrictive fluid management [21]. ARDS was defined according to the Berlin definition, using the PaO2/FiO2 ratio (Horowitz index) as marker for severity [22].

### Data analysis and statistics

Data were processed in a standardized script-based manner with the R software environment (version 4.1.2). For the final composition of Figures, Adobe Illustrator® was used (Version 24.3). A detailed description of the data preprocessing was previously described [23]. Numeric patient descriptives like age, and length of ICU stay (Table 1) are provided as mean and standard deviation (SD), scores or other quantitative variables were reported as median and interquartile range (IQR). Patients were separated according to their outcome (survivors and deceased). Analysis between groups were done with a linear model (aov: Fit an Analysis of Variance Model—stats package R). The longitudinal data was separated according to the length of the ICU stay into less than three days after admission (early), three to ten days after admission (intermediate), and more than ten days after admission (late). We assessed autonomic variability by calculating the standard deviation (SD) of HR (HR variability = HRV) and mean arterial pressure (MAP) per day (blood pressure variability = BPV). Statistical analysis of intergroup comparisons was performed for early, intermediate, and late timepoints by a mixed linear regression model for SD that was adjusted for age, sex and disease severity as measured by Sequential Organ Failure Assessment Score (SOFA-score). Intraindividual correlations were calculated with the mean value of each parameter per day by Pearson correlation. Subsequent between-group comparisons were performed using the signed negative logarithmic P-value of each correlation. Multiple comparisons were accounted for by FDR-adjustment. Data transformation, calculation, and visualization were done in R (version 4.1.2 main packages: tidyverse, ggpubr, readxl, openxlsx, aov, and lubridate). Significant results are indicated by * *P* < 0.05, **P < 0.01, ***P < 0.001, ****P < 0.0001.

**Table 1:** Patient characteristics

	Deceased	Survived	*P* value
	n = 124	n = 237	
Male	91 (73.4%)	149 (62.9%)	0.044
Age	64 (± 17.2)	59 (± 21.0)	< 0.001
ICU stay (days)	14.1 (± 18.7)	11 (± 27.3)	0.847
First symptoms (days)	10 (± 11.0)	6 (± 9.00)	0.014
Invasive breathing (hours)	286 (± 434)	322 (± 782)	0.286
Charlson Comorbidity Index (CCI)	1 (± 3.00)	1 (± 3.00)	0.481
ARDS-diagnosis	119 (96.0%)	125 (52.7%)	< 0.001
Mild	0 (0%)	8 (3.4%)	0.037
Moderate	9 (7.3%)	22 (9.3%)	0.516
Severe	110 (88.7%)	94 (39.7%)	< 0.001

## Results

### Recruitment and study cohort

The study was performed between 03/2020 and 11/2021. In this period 361 patients were treated in the ICU due to acute COVID-19 which were all included into our study (Table 1). 124 patients deceased during their ICU stay. Overall, more male than female patients were admitted to the ICU (66.4% male) and the proportion of male patients in the deceased group was significantly higher (73.4% male) compared with the surviving group (62.9% male, *P* = 0.045). Deceased patients were significantly older than survivors (*P* < 0.001). The Charlson Comorbidity Index (CCI) did not differ between those who survived and deceased patients (*P* = 0.481). Occurrence of severe ARDS was higher in the group of patients who deceased. The time spent with invasive mechanical ventilation was not significantly higher in the deceased group. The patients’ characteristics are summarized in Table 1.

### Longitudinal analysis of vital signs

**Fig. 1:**
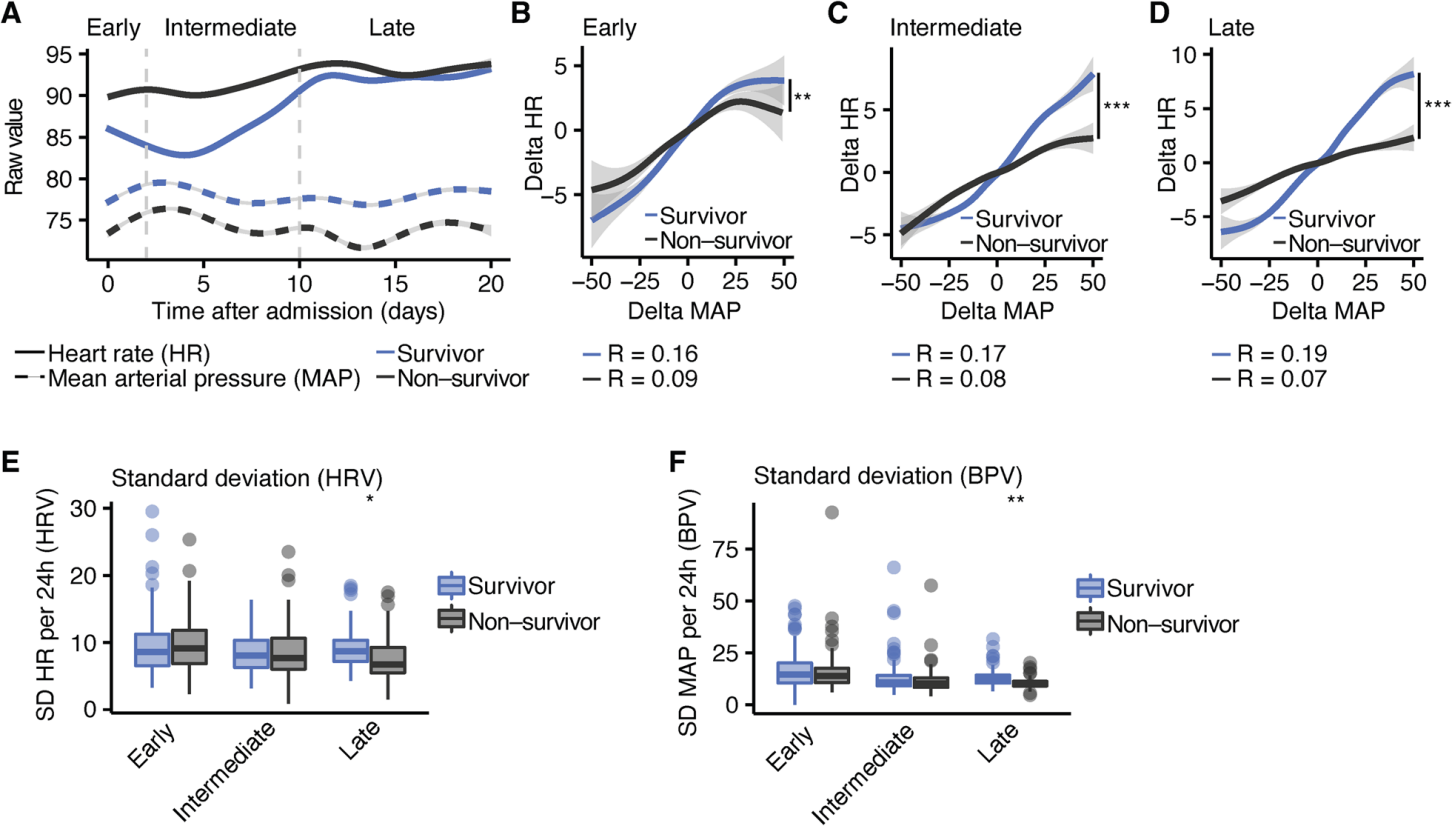
Heart rate (HR) and mean arterial pressure (MAP) over time in COVID-19 patients who were treated in ICU. **A** Time course of HR and MAP over time in patients with COVID-19 who were admitted to the ICU. The data was separated into less than three days (early), three to ten days (intermediate) and more than 10 days (late) after admission to ICU. **B**–**D** Correlation analysis between the change rate of the HR and the change rate of the MAP in early (**B**; *P* = 0.007), intermediate (**C**; *P* = 0.0002) and late (**D**; *P* = 0.00014) timepoints. **E** standard deviation of HR (HRV) of survivors and non-survivors at early, intermediate, and late timepoints after admission to the ICU. **F** and standard deviation of MAP (BPV) of survivors and non-survivors less than two days (early), two to ten days (intermediate) and more than 10 days (late) after admission to the ICU. Statistical analyses were performed by mixed linear model corrected for age and sex and FDR-adjustment for multiple comparisons. *P < 0.05, **P < 0.01, ***P < 0.001, ****P < 0.0001

First, the correlation between HR and MAP was analyzed (Fig. 1A shows longitudinal trajectories of HR and MAP). This revealed that changes of HR were significantly less correlated with changes in blood pressure in non-survivors as compared to survivors of COVID-19 in early (Fig. 1B; survivor r = 0.16 vs. non-survivor r = 0.09; *P* = 0.007), intermediate (Fig. 1C; survivor r = 0.17 vs. non-survivor r = 0.08; *P* = 0.0002) and late timepoints (Fig. 1D; survivor r = 0.19 vs. non-survivor r = 0.07; *P* = 0.0001). Next, we used the standard deviations per day of the heart rate (HRV) and blood pressure (BPV) as biomarkers for dysautonomia and compared survivors with deceased patients. Reduced HRV (Fig. 1E) and BPV (Fig. 1F) significantly predicted lethal outcome in the late phase (HRV: Estimate = − 1.5, SE = 0.4, *P* = 0.028); BPV: Estimate = − 2.3, SE = 0.6, *P* = 0.008) but not in the early or intermediate stages.

### Correlation of vital signs with laboratory data

**Fig. 2:**
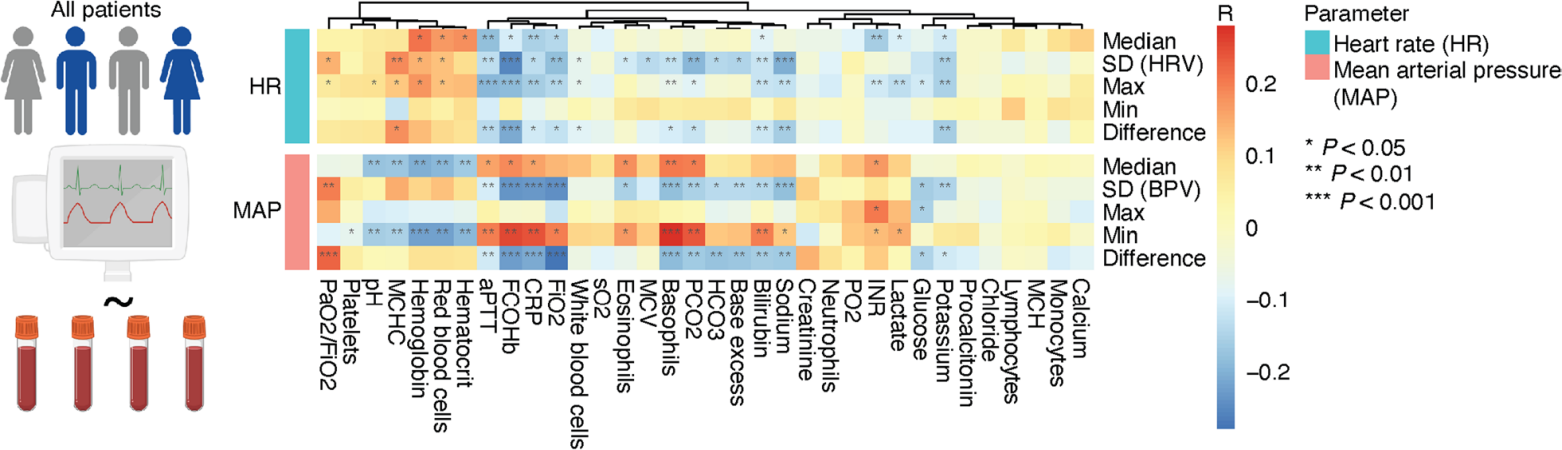
Correlation of heart rate (HR) and mean arterial pressure (MAP) with laboratory parameters. Correlation of median, standard deviation, maximal and minimal HR (top 5 rows) and MAP (bottom 5 rows) per day with indicated laboratory parameters across all patients. Colour shows correlation coefficient R. Pearson correlation was used for statistical analysis and FDR-adjustment was performed for multiple comparisons. *P < 0.05, **P < 0.01, ***P < 0.001

Next, we set out to investigate whether the HRV is differently modulated in survivors and non-survivors. First, we tested the hypothesis that increased inflammation and respiratory failure results in reduced HRV due to physiologic sympathetic adaptations [24]. Therefore, we performed correlation analyses of HRV and BPV with laboratory parameters across all patients (Fig. 2; all laboratory parameters are shown in the heatmap). Confirmatory, the inflammatory marker C-reactive protein (CRP) was significantly associated with decreased HRV and BPV. Increased pCO2, as a sign of respiratory failure negatively correlated with the HRV. In contrast, the HRV was increased in patients with a better blood gas exchange (measured by P/F or Horowitz index [25]) pointing towards a decreased HR modulation in systemic inflammation and respiratory failure.

**Fig. 3:**
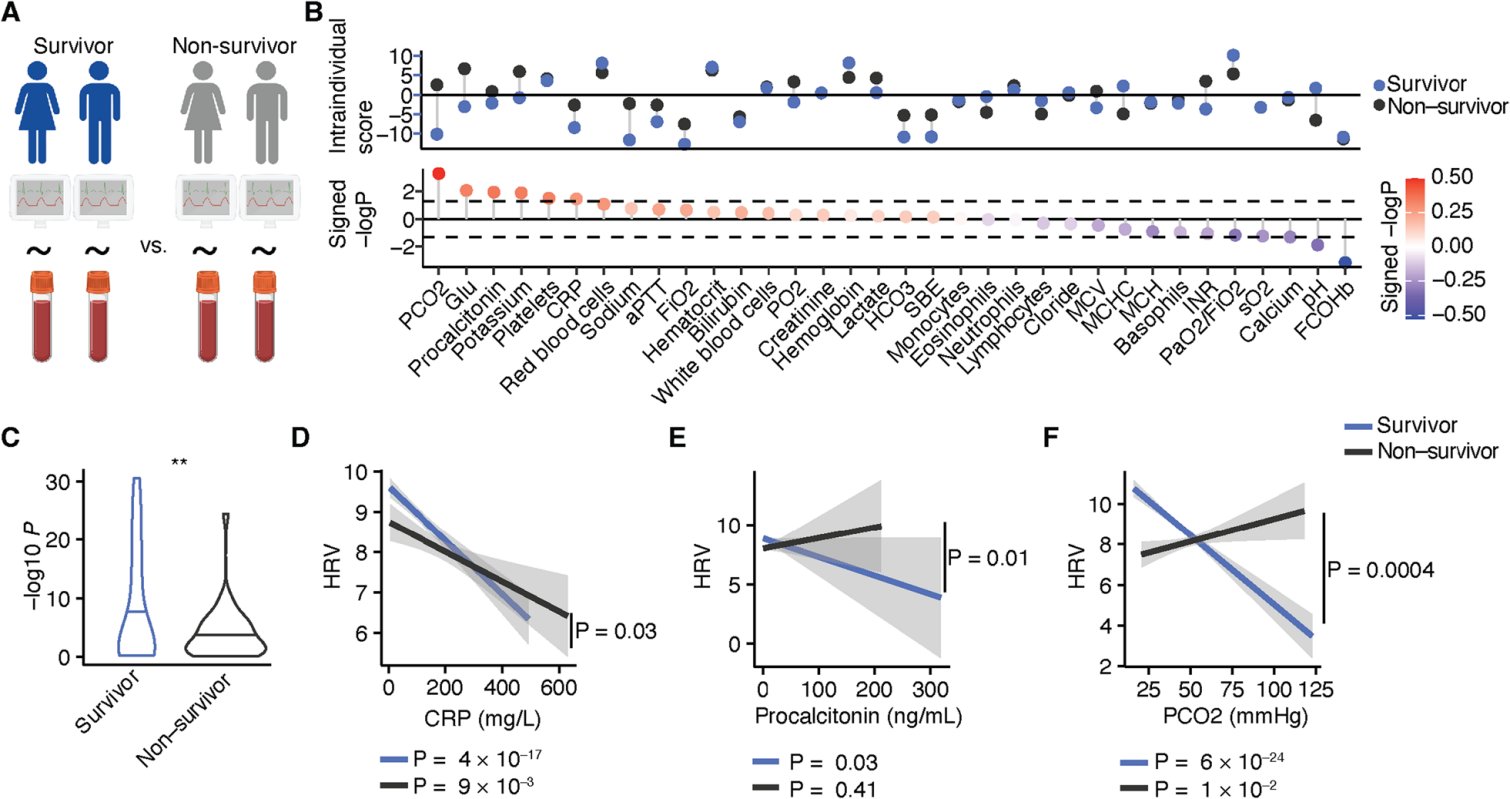
Heart rate variability (HRV) modulation dissociates from inflammation and respiratory failure in non-survivors. **A** and **B** Intraindividual correlation scores of HRV and each indicated laboratory parameter (top) and signed negative logarithmic *P* value (bottom). Dashed lines show *P* value of 0.05. **C** Comparison of all correlations of HRV with laboratory parameters between survivors and non-survivors. Unpaired t-test of − log10 *P* values was used for statistical comparison. **D**–**E** Correlation analyses of HRV with CRP (**C**), Procalcitonin (**D**) and blood carbon dioxide (**E**) separated by survivors and non-survivors. Pearson’s correlation analysis was performed. Group comparisons were performed by unpaired t-test of signed − log10 of the *P* value of intraindividual correlations. *P* values of correlation analyses and comparisons between survivors and non-survivors are depicted in the figure. *P < 0.05, **P < 0.01, ***P < 0.001

Finally, we investigated whether the HRV adaptations are impaired in non-survivors in comparison to survivors. Therefore, for each patient intraindividual correlations between HRV and all laboratory parameters across all timepoints on their stay on the ICU were calculated (Fig. 3A, B). First, we compared the overall significance of the calculated correlations between survivors and non-survivors (Fig. 3C). The HRV was significantly less modulated by other laboratory parameters in non-survivors in comparison to survivors (*P* = 0.03) supporting our hypothesis that HRV is less adaptative in non-survivors. When stratifying the correlations between HRV and each laboratory parameter, as expected a negative correlation of the HRV with CRP was found (survivor, mean R = − 0.2, SD = 1.3, *P* = 4e−17; non-survivor, mean R = 0.1, SD = 1.3, *P* = 9e−3). However, the correlation of HRV with CRP was significantly decreased in non-survivors (Fig. 3C; survivor vs non-survivor, *P* = 0.03). In addition, the HRV negatively correlates with procalcitonin (Fig. 3D) in survivors (mean R = − 0.1, SD = 1.2, *P* = 0.03) but not in non-survivors (mean R = 0.02, SD = 1.2, *P* = 0.41; survivor vs non-survivors, *P* = 0.01). Intriguingly, survivors showed a strong reduction of the HRV (Fig. 3E) with increasing blood carbon dioxide (mean R = − 0.1, SD = 1.3, *P* = 1e−24) whereas non-survivors exhibit an opposite regulation with a positive correlation of the HRV with blood carbon dioxide (mean R = 0.5, SD = 1.1, *P* = 0.01; Fig. 3F, survivor vs non-survivor, *P* = 0.0004) underlining the dysregulation of HR modulation in non-survivors of COVID-19 in comparison to survivors who were admitted to the ICU.

## Discussion

We hypothesized that ANS dysfunction contributed to mortality in COVID-19 patients who were admitted to the ICU during the first waves of the pandemic. To test our hypothesis, we used the HRV and BPV as clinical surrogate markers for vegetative dysfunction that were compared between survivors and non-survivors. Additionally, we performed correlation analyses with laboratory parameters that indicate respiratory failure and inflammatory activity in COVID-19. We observed that non-survivors showed a reduced correlation between HR and MAP in comparison to survivors and reduced HRV and BPV predicted lethal outcome. In addition, non-survivors adapted significantly worse to hypercapnia and systemic inflammation. These results point towards a contribution of ANS dysfunction to mortality in COVID-19 supporting current literature on its role in poor outcomes for the disease [26].

The profound disturbance of autonomic adaptation in non-survivors was evident in patients who spent over ten days in the ICU but not earlier. One possible explanation for the gap between disease onset and the observed ANS dysfunction is axonal damage and subsequent axonal degeneration that develop over several days [27]. Detecting ANS dysfunction in patients with severe COVID-19 is especially relevant since more therapies are becoming available for the treatment of COVID-19 patients and efficient distribution to those who need them most is crucial. Specific therapies include remdesivir, anti-interleukin-6 receptor antibodies, Janus kinase inhibitors [28, 29] as well as the microtubule disruptor sabizabulin [30] which has shown promising results in a recent phase II clinical trial in reducing mortality and respiratory failure. In addition to the potential prognostic value, our findings suggest that autonomic dysfunction itself increases mortality and could be an interesting target for therapeutic interventions for example in form of vagal nerve stimulation or adaptation of management of fluid therapy, mechanical ventilation, or use of vasopressors.

Invasive breathing with positive pressure increases the intrathoracic pressure changes between respiratory cycles and thereby causes a higher sympathetic tonus augmenting HRV [31]. Therefore, we cannot exclude an effect of positive pressure ventilation on our observed HRV differences between survivors and non-survivors. However, contradictory to the HRV increase in mechanically ventilated patients, we observed a decrease of HRV in non-survivors who were mostly under respiratory support and suffered significantly more often from severe ARDS. This underlines that the decrease of HRV is not explained by mechanical ventilation but most likely shows autonomic dysfunction in COVID-19 non-survivors. Moreover, models used in the presented study were adjusted for disease severity and we observed different HR modulation by inflammatory markers in survivors and non-survivors. Interestingly, reduced HRV is associated with the failure of weaning after mechanical ventilation [32], underlining a potential prognostication of reduced HRV as surrogate for disease outcome in severe COVID-19.

Some limitations of this study must be addressed. Firstly, due to the limited number of patients and data resolution, it was not possible to build models that would allow predicting severe disease courses for every single patient based on autonomic dysfunction which will be a goal for the future. In addition, it cannot be concluded that autonomic dysfunction is specific to SARS-CoV-2 infection from the results of the study since no comparison with other viral infections and critically ill patients was performed. Autonomic dysfunction is a well-established predictor of unfavorable outcomes on ICU [33, 34] and has been described in the context of other viral infections as well, of which HIV, HTLV1, herpes viruses, tick-borne encephalitis virus, and West Nile virus are the most common ones [35]. Interestingly, all these viruses frequently infect the central and peripheral nervous systems. This makes our approach of measuring HRV and BPV and correlating it with laboratory parameters for inflammation and respiratory failure potentially usable for other infectious diseases that can cause sepsis since it is simply, non-invasive, and cost-effective. Furthermore, the study cannot explain mechanistically how autonomic dysfunction arises and which structures of the ANS are involved. This will be important to clarify in future studies since it is relevant for the development of targeted treatments. Thus, autonomic dysfunction could be explained by direct viral infection of structures of the autonomic nervous system. This hypothesis is supported by the findings that structures of the ANS such as the vagal nerve express receptors for SARS-CoV-2 entry. Alternatively, activation of the immune system by the virus and an overshooting immune response could damage the ANS due to the formation of autoantibodies or as a result of the cytokine storm.

Furthermore, the study is limited by its mono-centric design. However, due to close clinical and laboratory monitoring, longitudinal patient data could be correlated with outcomes. In the timeframe of inclusion for this study, the only SARS-CoV-2 variants that existed in Germany were the alpha, beta and delta variants (www.rki.de). At the end of 2021, the omicron variant emerged which rapidly evolved into the most frequent SARS-CoV-2 variant which is not included in the cohort. In contrast to the previous SARS-CoV-2 variants, omicron shows less severe disease courses with less involvement of the CNS [36]. Additionally, the number of patients admitted to the ICU due to omicron infection is considerably smaller than for the previous variants [37]. Dysautonomia in patients with omicron infection needs to be addressed in future studies.

## Conclusions

In summary, dysautonomia is a common and severe complication in critically ill patients with acute COVID-19 and reduced BPV and HRV predicted lethal outcome. Accordingly, the physiologic sympathetic HRV adaptation to inflammation and respiratory failure were decreased in non-survivors implying an impaired autonomous regulation in critically ill COVID-19 patients. Prospective studies should focus on the clinical implications and prognostic value of dysautonomia to identify patients at risk for lethal disease outcome.

## Data Availability

Data are available from the corresponding author, upon reasonable request.

## Abbreviations

ANS: Autonomic nervous system
CI: Confidence interval
COVID-19: Corona virus disease 2019
CRP: C-reactive protein
GCS: Glasgow Coma Scale
pCO2: Arterial carbon dioxide partial pressure
SARS-CoV-2: Severe acute respiratory syndrome coronavirus type 2
ACE2: Angiotensin-converting enzyme 2
ICU: Intensive Care Unit
TMPRSS2: Transmembrane, protease serine subtype 2
NRPI1: Neuropilin 1
HR: Heart rate
MAP: Mean arterial pressure
SOFA: Sequential organ failure assessment score
CCI: Charlson Comorbidity Index
PCR: Polymerase chain reaction
PaO2: Partial arterial oxygen pressure
FiO2: Fraction of inspired oxygen
SE: Standard error
